# Nucleomorph and plastid genome sequences of the chlorarachniophyte *Lotharella oceanica*: convergent reductive evolution and frequent recombination in nucleomorph-bearing algae

**DOI:** 10.1186/1471-2164-15-374

**Published:** 2014-05-15

**Authors:** Goro Tanifuji, Naoko T Onodera, Matthew W Brown, Bruce A Curtis, Andrew J Roger, Gane Ka-Shu Wong, Michael Melkonian, John M Archibald

**Affiliations:** Department of Biochemistry and Molecular Biology, Canadian Institute for Advanced Research, Integrated Microbial Biodiversity Program, Dalhousie University, Halifax, Nova Scotia B3H 4R2 Canada; Faculty of life and environmental sciences, University of Tsukuba, Tsukuba, Ibaraki 305-8577 Japan; Department of Biological Sciences, Mississippi State University, Mississippi State Mississippi, 39762 USA; Department of Biological Sciences, University of Alberta, Edmonton, AB T6G 2E9 Canada; Department of Medicine, University of Alberta, Edmonton, AB T6G 2E1 Canada; BGI-Shenzhen, Beishan Industrial Zone, Yantian District, Shenzhen 518083 China; Department of Botany, Cologne Biocenter, University of Cologne, Cologne, 50674 Germany

**Keywords:** Nucleomorph, Genome reduction, Chlorarachniophytes, Cryptophytes, Endosymbiosis, Phylogenomics

## Abstract

**Background:**

Nucleomorphs are residual nuclei derived from eukaryotic endosymbionts in chlorarachniophyte and cryptophyte algae. The endosymbionts that gave rise to nucleomorphs and plastids in these two algal groups were green and red algae, respectively. Despite their independent origin, the chlorarachniophyte and cryptophyte nucleomorph genomes share similar genomic features such as extreme size reduction and a three-chromosome architecture. This suggests that similar reductive evolutionary forces have acted to shape the nucleomorph genomes in the two groups. Thus far, however, only a single chlorarachniophyte nucleomorph and plastid genome has been sequenced, making broad evolutionary inferences within the chlorarachniophytes and between chlorarachniophytes and cryptophytes difficult. We have sequenced the nucleomorph and plastid genomes of the chlorarachniophyte *Lotharella oceanica* in order to gain insight into nucleomorph and plastid genome diversity and evolution.

**Results:**

The *L. oceanica* nucleomorph genome was found to consist of three linear chromosomes totaling ~610 kilobase pairs (kbp), much larger than the 373 kbp nucleomorph genome of the model chlorarachniophyte *Bigelowiella natans*. The *L. oceanica* plastid genome is 71 kbp in size, similar to that of *B. natans*. Unexpectedly long (~35 kbp) sub-telomeric repeat regions were identified in the *L. oceanica* nucleomorph genome; internal multi-copy regions were also detected. Gene content analyses revealed that nucleomorph house-keeping genes and spliceosomal intron positions are well conserved between the *L. oceanica* and *B. natans* nucleomorph genomes. More broadly, gene retention patterns were found to be similar between nucleomorph genomes in chlorarachniophytes and cryptophytes. Chlorarachniophyte plastid genomes showed near identical protein coding gene complements as well as a high level of synteny.

**Conclusions:**

We have provided insight into the process of nucleomorph genome evolution by elucidating the fine-scale dynamics of sub-telomeric repeat regions. Homologous recombination at the chromosome ends appears to be frequent, serving to expand and contract nucleomorph genome size. The main factor influencing nucleomorph genome size variation between different chlorarachniophyte species appears to be expansion-contraction of these telomere-associated repeats rather than changes in the number of unique protein coding genes. The dynamic nature of chlorarachniophyte nucleomorph genomes lies in stark contrast to their plastid genomes, which appear to be highly stable in terms of gene content and synteny.

**Electronic supplementary material:**

The online version of this article (doi:10.1186/1471-2164-15-374) contains supplementary material, which is available to authorized users.

## Background

Endosymbiosis has been a driving force in the evolution of eukaryotic cells. All known eukaryotes possess mitochondria (or mitochondrion-derived organelles), which evolved from an ancestor of modern-day alpha-proteobacteria [1, 2]. Plastids, the light-gathering organelles of plants and algae, have evolved in several different ways [3, 4]. The plastids of green, red and glaucophyte algae are believed to have evolved from a single “primary” endosymbiotic event that occurred between a eukaryotic host and a cyanobacterial endosymbiont. Subsequently, the primary plastids of red and green algae spread to other eukaryotic lineages by “secondary” endosymbiosis, e.g., in the haptophytes, stramenopiles and euglenophytes. Genome size reduction is a commonly seen phenomenon in endosymbiosis. Plastid and mitochondrial genomes are typically <5% of the size of the bacterial genomes from which they are believed to have evolved [5, 6] and in the case of secondary endosymbiosis the nucleus of the eukaryotic endosymbiont is usually lost entirely [3, 4]. This massive genome reduction is due to the combined effects of endosymbiotic gene transfer (EGT) and the outright loss of genes presumed to be unnecessary for an endosymbiotic lifestyle [5–7]. More than 1,000 organelle-targeted proteins, encoded by the host nucleus, are necessary for the proper functioning of modern-day plastids and mitochondria [8].

Nucleomorphs are residual secondary endosymbiont nuclei found in two different eukaryotic algal lineages, the chlorarachniophytes and cryptophytes [9]. These unusual organelles exist between the second and third envelope membranes of their plastids. This space is known as the periplastidial compartment (PPC), which corresponds to the cytosol of the engulfed eukaryotic endosymbiont. Molecular phylogenetic analyses have shown that the nucleomorph and plastid of chlorarachniophytes are derived from endosymbiotic green algae, whereas red algae gave rise to the nucleomorph and plastid in cryptophytes [10–12]. Nucleomorph genomes are highly reduced: at present the observed size range is between 0.33 and 1 megabase pairs (Mbp) [13–15]. Despite their independent origins, the nucleomorph genomes of both cryptophytes and chlorarachniophytes exhibit common structural features such as the presence of three linear chromosomes and sub-telomeric ribosomal RNA (rRNA) operons [9, 13, 14, 16]. These similarities suggest that both endosymbiont genomes have been subjected to similar reductive pressures during the process of secondary endosymbiosis.

Nucleomorph genomes have been completely sequenced in one chlorarachniophyte, *Bigellowiela natans*[17], and four cryptophytes, *Guillardia theta*[18], *Hemiselmis andersenii*[19]*, Cryptomonas paramecium*[20] and *Chroomonas mesostigmatica*[21]. The number of protein genes encoded by the nucleomorph genomes examined thus far is between 300 and 500 and gene density is high, in some cases approximately one gene per kbp. Protein sequence lengths and intergenic spacer regions are also reduced compared to those of free-living algae [19–21]. As in mitochondria, plastids, and some endosymbionts and parasites, nucleomorph genomes exhibit a highly biased A + T content (ca. 75%) [9, 22–26]. Most of the proteins encoded by nucleomorph genomes are predicted to be involved in housekeeping functions such as translation and transcription [17–21]. Only a small number of genes for plastid-associated proteins are present (17 in the chlorarachniophyte *B. natans* and 18–31 in cryptophytes). Tanifuji *et al.*[20] found that the sequenced nucleomorph genomes of cryptophytes and chlorarachniophytes share a similar set of core proteins, suggesting convergent patterns of gene retention in these independently reduced genomes. Overall, chlorarachniophyte and cryptophyte nucleomorph genomes are similar in terms of size, structure and gene content, despite having evolved from different algal endosymbionts.

Perhaps the most striking difference between chlorarachniophyte and cryptophyte nucleomorph genomes is the number of spliceosomal introns. While chlorarachniophyte nucleomorph genomes are intron-rich (*B. natans* has 852 of them), few or no introns are found in cryptophyte nucleomorph genomes [9, 17–21, 27]. Nucleomorph introns in the chlorarachniophytes *B. natans* and *Gymnochlora stellata* are tiny (18–24 bp) compared to those in green algae [17, 27]. In the green algae *Chlamydomonas reinhardtii* and *Ostreococcus tauri*, for example, mean intron lengths are 373 and 103 bp, respectively [28, 29]. Comparison of intron positions in the *B. natans* nucleomorph genome to green algae (e.g., *C. reinhardtii*) and plants suggest that the nucleomorph introns were acquired from the green algal nucleus that gave rise to the nucleomorph [17, 30]. The radical intron size reduction in chlorarachniophyte nucleomorph genomes appears to have taken place prior to the divergence of the modern-day species that have been examined [27].

Although knowledge of nucleomorph genome evolution is accumulating, progress is hampered by the existence of only a single chlorarachniophyte nucleomorph genome sequence. The true diversity of nucleomorph genome structure and coding capacity in chlorarachniophytes is at present unclear. In order to gain insight into the patterns and processes of reduction within and between chlorarachniophyte and cryptophyte nucleomorph genomes, additional sequences from chlorarachniophytes are necessary. Plastid genome sequences are also important pieces of the puzzle. Complete plastid genomes have been sequenced for three cryptophytes, *G. theta*[31], *Rhodomonas salina*[32] and *C. paramecium*[33], but only a single chlorarachniophyte plastid genome sequence is presently available, that of *B. natans*[12].

Here we present complete nucleomorph and plastid genome sequences for the chlorarachniophyte *Lotharella oceanica* and compare them to their counterparts in the model chlorarachniophyte *B. natans*. The results suggest that recombination is an important factor driving the shrinkage and occasional expansion of nucleomorph genomes in chlorarachniophytes and perhaps cryptophytes. We have also carried out a phylogenetic analysis of nucleomorph and plastid proteins in order to gain insight into the origin of the chlorarachniophyte secondary endosymbiont.

## Results and discussion

### *Lotharella oceanica* nucleomorph and plastid genome sequences

**Figure 1:**
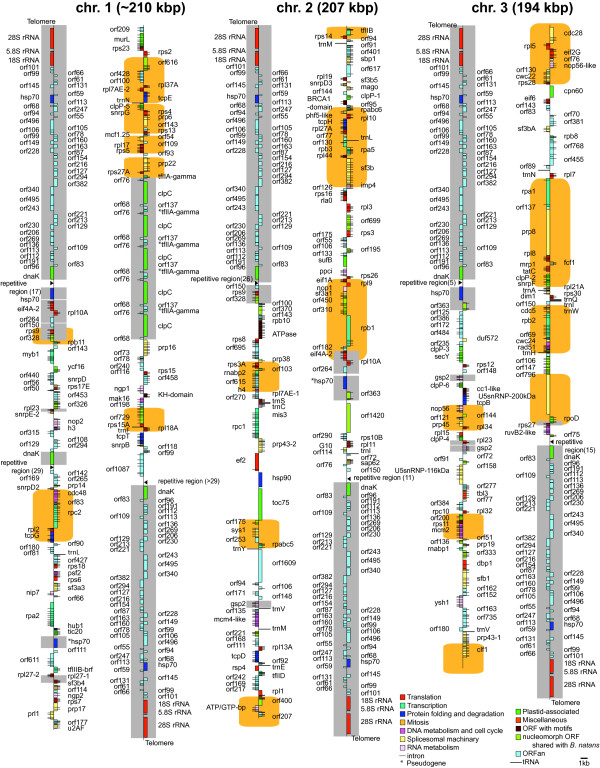
**Physical map of the** ***Lotharella oceanica*** **nucleomorph genome.** The genome is ~610 kbp in size with three chromosomes, shown artificially broken at their midpoint. Annotated genes are colored according to the functional categories shown in the lower right. The exact number of tandem repeats containing the ClpC and tfIIa-gamma genes on chromosome I is not known but was estimated to be at least five. Orange boxes indicate regions syntenic with the *Bigelowiella natans* nucleomorph genome (see text). Gray boxes show multi-copy regions. Genes mapped on the left side of each chromosome are transcribed bottom to top and those on the right, top to bottom.

The *L. oceanica* nucleomorph genome consists of three linear chromosomes totaling ~610 kbp, which is 240 kbp larger than *B. natans*, the first complete nucleomorph genome sequenced for a chlorarachniophyte (Figure 1 and Table 1). The *L. oceanica* chromosomes are ~210,000, 207,543 and 194,115 bp in size. On chromosome I, an internally repeated region consisting of at least five tandem repeats containing ClpC and tfIIa-gamma genes and three additional ORFs was identified (the repeat number was estimated by considering the sequence coverage depth in this region) (Figure 1). Because of this repeat, the exact size of chromosome I is unclear. However, our assembly-based chromosome size predictions are consistent with previous estimates of ~210, ~205, and ~195 kbp obtained by pulsed-field gel electrophoresis [13].

**Table 1:** **Summary of chlorarachniophyte nucleomorph and plastid genomes**

**Nucleomorph**	***L. oceanica***	***B. natans***
Total genome size (bp)	~611,658	372,879
chr. 1 size (bp)	~210,000	140,598
chr. 2 size (bp)	207,543	134,144
chr. 3 size (bp)	194,115	98,137
G + C content (%)	33.0 (24.0*)	28.5 (25.1*)
# of introns	1,011	951**
# of tRNAs	19	19
Average intergenic spacer size (bp)	147.4(140.9*)	82.1
# of protein genes	610	284**
# of non-redundant protein genes	348	283
**Plastid**	***L. oceanica***	***B. natans***
Total genome size (bp)	70,997	~69,000
# of protein genes	60	61
G + C content (%)	30.6	30.2
# of tRNAs	28	31

*Calculated with subtelomeric regions excluded.

**Numbers updated from original publication (Gilson et al. [17]).

The G + C content of the three *L. oceanica* nucleomorph chromosomes is 33.6%, 32.8%, and 32.7% for I, II, and III, respectively. However, ~35 kbp sub-telomeric regions repeated on each of the six chromosome ends were found to be of much higher G + C content, 49.4% (see below). Excluding the sub-telomeric regions, the G + C content is 25.3, 23.9, and 22.8% for chromosomes I, II, and III, similar to that in the *B. natans* nucleomorph genome (25.1%) as well as the four cryptophyte nucleomorph genomes sequenced to date (25.2%-26.4%) [9, 17–21].

**Figure 2:**
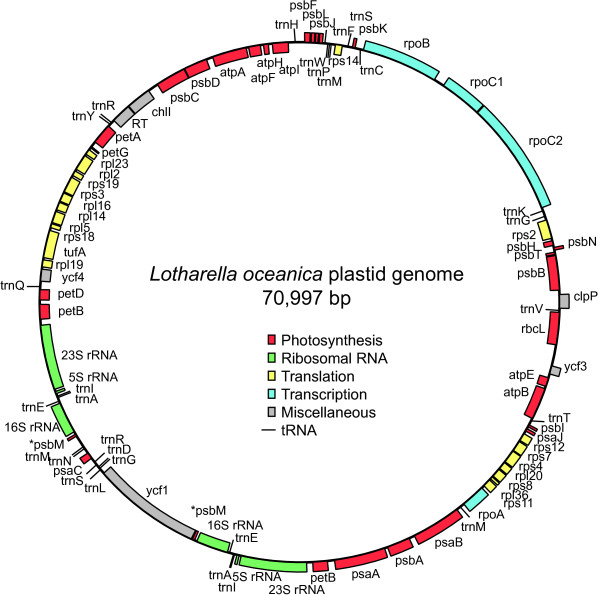
**Circular physical map of the plastid genome of** ***Lotharella oceanica.*** The 70,997 bp genome contains inverted rRNA operons, 60 predicted protein genes, and 28 tRNA genes. Genes shown on the outside of the circle are transcribed clockwise. Annotated genes are colored according to the functional categories shown in the center.

The plastid genome of *L. oceanica* was found to be circular and 70,997 bp in size, encoding 60 proteins (two pseudogenes) and 28 tRNAs, plus 6 rRNAs in inverted repeats (Figure 2 and Table 1). The G + C content is 30.6%. The genome is highly similar to the *B. natans* plastid genome in size, gene order, G + C content and structure, albeit with a few exceptions: 1) a psaA gene is missing from one of the two ends of the inverted repeats, and a petD gene is missing from the other end; 2) the position of petD and petB are switched; 3) a gene encoding a putative reverse transcriptase was found in *L. oceanica* between the chlI and petA genes, implying the existence of a group II intron in *L. oceanica*. However, an ORF was not detectable in the vicinity of the reverse transcriptase-encoding region (Figure 2). The *L. oceanica* plastid genome contains three small inverted repeats. One consists of a 28 bp sequence located between the psbJ and tRNA-Phe genes, and two inverted repeat pairs consisting of 76 bp and 93 bp are located side by side between atpI and psbE*.* The region between the atpI and psbE genes could not be sequenced in *B. natans*, presumably due to extensive secondary structure. We were successful in determining the DNA sequence of the corresponding region in the *L. oceanica* plastid genome. However, its sequence characteristics do not provide further insight into the possibility that it is an origin of replication site, as was suggested for *B. natans*[12]. The unusual rRNA operon inversion seen in *B. natans*, in which the small and large subunit rRNA genes are on the opposite strand, is also present in the *L. oceanica* plastid genome.

### Synteny, gene content and intron evolution in chlorarachniophyte nucleomorph genomes

The structure and coding capacity of the nucleomorph genomes in *L. oceanica* and *B. natans* are summarized in Table 1 and Additional file 1. 610 protein genes were predicted in the *L. oceanica* nucleomorph genome; this number is almost twice as many as in *B. natans*. However, this is mainly due to a total of 258 predicted ORFs with no introns in the ~35 kbp sub-telomeric regions. In fact, the number of non-redundant protein genes (counting each multicopy gene as one) is 348, 65 more than in *B. natans* (283) (Table 1 and Additional file 1). Since the sub-telomeric region of each chromosome end shows extremely low levels of gene expression compared with internally located genes, it is unclear if the sub-telomeric ORFs are real protein-coding genes (see below). In addition, the total number of introns in both genomes is similar, with 1,011 in *L. oceanica* and 951 in *B. natans* (see following section). There are 19 tRNA genes in both genomes, but with a few differences in their content compared to each other. Bona fide snRNAs were not found in the *L. oceanica* nucleomorph genome.

The average intergenic spacer size in the *L. oceanica* genome is 147.4 bp, which is significantly higher than in *B. natans* (82.1 bp) (supported by a T-test (*p* < 0.001) (Table 1)). The average length of all predicted proteins in the *L. oceanica* genome (247.2 amino acids) was found to be smaller than that in *B. natans* (326.8) (*p* < 0.001). However, no significant difference (*p* > 0.05) was seen in the average length of 166 shared proteins (excluding ORFs with identifiable protein motifs and unique ORFs found in nucleomorph genomes), with an average of 334.4 amino acids in *L. oceanica* and 343.1 in *B. natans*.

The *L. oceanica* nucleomorph chromosomes were found to contain a repetitive sequence consisting of a 36 nt repeat located between all of the ~35 kbp sub-telomeric regions and the internal single-copy regions (Figure 1; between 5 and >29 repeats of the following sequence: RTAYCTRGTTRCCTTATCGTATGCCATGGCTTTATC). Repetitive sequences were found in the nucleomorph genome of the cryptophyte *C. mesostigmatica*[21], but in that case they consisted of A-T-rich simple repeats such as [TTA]_n_ located in the ITS between the 5S and 28S rDNA, and [AT_4–5_]_14_ and [TA_2_GA_2_TA_5_]_4–25_ in intergenic spacers of the sub-telomeric repeats. A long homopolymer of [A/T]_24–37_ was also found in several sites within 28S rDNA in *C. mesostigmatica*. In contrast, the repetitive sequences in *L. oceanica* were less A-T rich and less variable in sequence. Intriguingly, each 36 nt repeat in *L. oceanica* has a potential initiation codon in the same reading frame as the gene for the molecular chaperone dnaK, which is of cyanobacterial origin and presumed to be targeted to the plastid. However, the transit peptide prediction for the repetitive sequence, as well as the region immediately upstream of the predicted dnaK start codon, did not identify a stretch of amino acids predicted to target the protein to the plastid. On the other hand, an additional dnaK gene located internally on chromosome I, was predicted to encode a transit peptide using the ChloroP [34] and TargetP [35] programs.

Genome synteny has been investigated previously in four completely sequenced cryptophyte nucleomorph genomes [19–21]. In *L. oceanica* and *B. natans* we identified 17 short blocks of synteny (14 blocks using the same criteria used by Moore *et al*. [21]) (Figure 1). In contrast, the plastid genomes in the two chlorarachniophytes showed almost the same gene order. A similar pattern is seen in cryptophytes. The plastid genomes of the photosynthetic species *G. theta* and *R. salina* are completely syntenic, and while the non-photosynthetic species *C. paramecium* contains one rearranged region around the ribosomal operon in its plastid genome, the overall level of synteny is still high [31–33]. This suggests that recombination is much more frequent in the nucleomorph genomes of both cryptophytes and chlorarachniophytes than in the plastid genomes (refer to the following section for further discussion). In sum, the nucleomorph genomes of *B. natans* and *L. oceanica* differ remarkably in terms of the number of predicted proteins, structure and synteny, while their plastid genomes have changed little since the two organisms diverged from a common ancestor.

### Sub-telomeric repeats and secondary expansion of the *L. oceanica* nucleomorph genome

**Figure 3:**
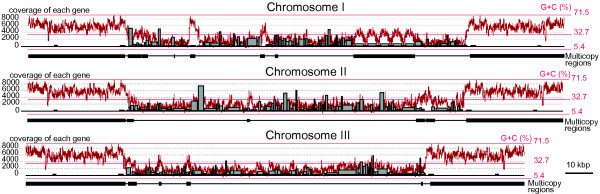
**G + C content and gene expression in the** ***Lotharella oceanica*** **nucleomorph genome.** Gray boxes indicate gene expression levels corresponding to RNA-Seq coverage depth of each gene. Red lines show the G + C levels on the chromosomes, which were captured from Artemis genome annotation software with the default setting. The black boxes under the graph indicate multicopy regions.

The most unexpected finding in the *L. oceanica* nucleomorph genome was the presence of large, nearly identical (with only 3 nucleotide differences) ~35 kbp sub-telomeric repeats on each of the six chromosome ends (Figure 1). As mentioned above, these repeats have a G + C content (49.4%) that is much higher than the internal regions of the chromosomes, which are ~25% G + C (Figure 3). Lane *et al.*[36] reported a ‘high’ G + C content in the short sub-telomeric region (830–4,030 bp) between the rDNA operon and the nearby ubc4 gene in the cryptophyte nucleomorph genomes of *G. theta*, *Hanusia phi* and *Proteomonas sulcata*. Regions of ~50% G + C, including the rDNA operons themselves, also exist on at least four of the six chromosome ends in completely sequenced cryptophyte nucleomorph genomes [9]. However, in these species the repeats are relatively short with lengths ranging from 7–12 kbp (including 6–7.6 kbp of rDNA).

Also remarkable are the protein genes located within the nucleomorph sub-telomeric repeats of *L. oceanica*. Of the 45 protein genes predicted in this region, only two show obvious sequence similarity to known proteins (dnaK and hsp70) (as in previous studies, potential genes were defined as being >150 nucleotides in length from an initiator methionine codon). Interestingly, transcriptome analyses of *L. oceanica* showed that the level of transcription of these 43 unknown genes in sub-telomeric repeats was extremely low (the mean RNA-Seq coverage of the 43 ORFs was 0.536; 26/46 ORFs show no coverage at all), almost 2,500 times lower than the average expression level for the protein genes in single-copy regions of the chromosomes (Figure 3). It is therefore possible that the 43 ORFs lacking obvious functions in the *L. oceanica* sub-telomeric regions are in fact not real protein genes. A recent comprehensive analysis of nucleomorph gene expression in *G. theta, C. mesostigmatica, C. paramecium* and *B. natans* showed that as a whole, nucleomorphs exhibit high transcription levels, with >97% of these four nucleomorph genomes being transcribed into mRNA, including non-coding regions [37]. Therefore, the extremely low transcription levels of the ORFs residing within the ~35 kb sub-telomeric regions of the *L. oceanica* genome is unusual, and consistent with them being spurious ORFs. The completely sequenced nucleomorph genomes of *G. theta, C. mesostigmatica, C. paramecium* and *B. natans* also contain sub-telomeric repeats, but they are much shorter than those in *L. oceanica*, and we were unable to obtain a clear picture of the extent to which they are expressed.

What is the biological significance of the long sub-telomeric repeat regions in *L. oceanica*? Gene conversion via genetic recombination is the most likely explanation for the maintenance of the near-identical rDNA sequences at the nucleomorph chromosome ends and such a process presumably acts to homogenize the adjacent sub-telomeric region as well. It seems significant that there are short repetitive sequences located next to the ~35 kbp sub-telomeric regions in *L. oceanica*, which show variable numbers of sequence units (x5 - >29 repeats, 180 - ~1,000 bp) (Figure 1). These heterogeneous repetitive sequences might be mediators of genetic recombination within and between nucleomorph chromosomes. Also, the elevated G + C content of the sub-telomeric regions in the *L. oceanica* nucleomorph genome is consistent with the phenomenon of GC-biased gene conversion, which has been demonstrated in other genomes such as those of mammals [38, 39].

Investigation of the hsp70 gene in *L. oceanica* provided insight into nucleomorph genome dynamics. No fewer than 10 copies of hsp70 were found in the genome: an ‘internal’ gene on chromosome I, another on chromosome II, six copies within the sub-telomeric repeats and two pseudogenes on chromosomes I and III (Figure 1). The sub-telomeric hsp70 genes have a G + C content of 44.1%, higher than the other isoforms (37.3% and 35.0%). Homologous recombination events have presumably occurred at the nucleomorph chromosome ends, resulting in hsp70 genes whose sequences are homogenized by gene conversion. The G + C content of the hsp70 genes located internally on chromosomes I and II are still higher than the average G + C content (~25%, excluding sub-telomeric regions). This is the case for other internally located multicopy regions as well (Figure 3). These observations suggest that gene conversion may be homogenizing multicopy genes throughout the *L. oceanica* nucleomorph genome, not just those residing in sub-telomeric regions. Such a process could result in the loss of some or all of the sub-telomeric repeat regions; homologous regions could disappear by unequal crossing over between sister chromatids. In cryptophytes, evidence for active recombination in nucleomorph genomes has come from investigations of *C. paramecium* and *H. anderseniii*, where complete rDNA operons have been lost from two and three of the six chromosome ends, respectively (only 5S ribosomal rDNA regions remain) [19, 20].

In order to gain further insight into the dynamic nature of nucleomorph chromosomes in *L. oceanica*, we examined the evolution of hsp70, a multi-copy gene present at least once on all three chromosomes and whose protein product is amenable to phylogenetic analysis. The three main types of hsp70 genes/proteins in *L. oceanica* were found to be monophyletic in phylogenetic trees and to branch sister to the *B. natans* nucleomorph hsp70, which is not sub-telomeric in its location (Additional file 2). This result is consistent with the idea that gene duplication occurred sometime after *L. oceanica* and *B. natans* diverged from one another. Alternatively, *B. natans* may have possessed multiple hsp70 genes in its nucleomorph genome in the past, as does *L. oceanica* today, but lost them, perhaps due to recombination. On balance, however, it seems likely that while nucleomorph genomes are highly reduced, active recombination at the sub-telomeric repeats can serve to expand the genome over evolutionary time.

Previous nucleomorph comparative genomic investigations revealed the existence of numerous blocks of synteny (more than three homologous genes in the same order) among the cryptophyte genomes of *G. theta*, *H. andersenii* and *C. paramecium*, some of which are in the range of ~25-45 kbp. However, the genome of the cryptophyte *C. mesostigmatica* was found to be much more fragmented compared to the other three, despite the fact that *C. mesostigmatica* and *H. andersenii* are specifically related to one another [21]. Moore et al. [21] showed that in *C. mesostigmatica*, the average number of genes in each syntenic block compared to *G. theta*, *H. andersenii* and *C. paramecium* is 6.7-9.0, whereas it is 9.4-10.9 when these three cryptophyte species are compared to one another. Compared to their plastid genomes, which show an extremely high level of gene synteny, the nucleomorph genomes of *L. oceanica* and *B. natans* are highly scrambled. We identified 14 nucleomorph syntenic blocks (using the same criteria as Moore *et al.*[21]) and the average number of genes in these regions was found to be 5.5, even smaller than the level of synteny seen between *C. mesostigmatica* and the other three cryptophytes nucleomorph genomes. Lane *et al*. [19] suggested that the high level of synteny in cryptophyte nucleomorph genomes is due to relatively low rates of recombination in internal regions. Why might the nucleomorph genomes of *L. oceanica* and *C. mesostigmatica* possess somewhat smaller blocks of synteny compared to the other nucleomorph genomes? Although unrelated to one another, these two nucleomorph genomes are similar in their possession of multicopy regions, repetitive sequence elements and somewhat larger genome sizes. Therefore, it is possible that in addition to homologous recombination at the sub-telomeric regions, homologous and non-homologous recombination in internal portions of the chromosomes have occurred along the entire lengths of the chromosomes in *L. oceanica* and *C. mesostigmatica*. The significantly larger mean intergenic spacer size in *L. oceanica* (147.4 bp) compared to *B. natans* (82.1 bp) also supports the idea of more frequent internal recombination; in regions where gene density is lower, recombination events would be less likely to disrupt essential genes. Compared to other cryptophytes, the *C. mesostigmatica* nucleomorph genome shows a somewhat larger average intergenic spacer size as well [21]. A more detailed picture of the impact of recombination on genome structure in chlorarachniophyte nucleomorphs will require genomic data from additional strains and species; with only two sequences in hand, it is unclear which of the two genomes—that of *L. oceanica* or *B. natans*—is more recombinagenic.

### Convergent nucleomorph genome evolution in cryptophytes and chlorarachniophytes

The *L. oceanica* nucleomorph genome contains 610 predicted protein genes. Of the 348 non-redundant protein genes, 160 were categorized as proteins with predicted functions shared with other eukaryotes under previously proposed criteria [20]. 17 of 348 non-redundant protein genes had homologs in cyanobacteria and were designated ‘plastid-associated’. 171 protein genes were considered to be ‘nucleomorph ORFans’ (nORFans), that is, nucleomorph proteins with unknown functions (most of these proteins show no sequence similarity to any known protein, in nucleomorphs or other genomes; see Additional file 1). However, 43 of these nORFans reside within the sub-telomeric repeats (43 nORFans × 6 repeats), and thus account for 25% of the non-redundant nORFan genes. These genes were removed from our comparative genomic investigations.

**Figure 4:**
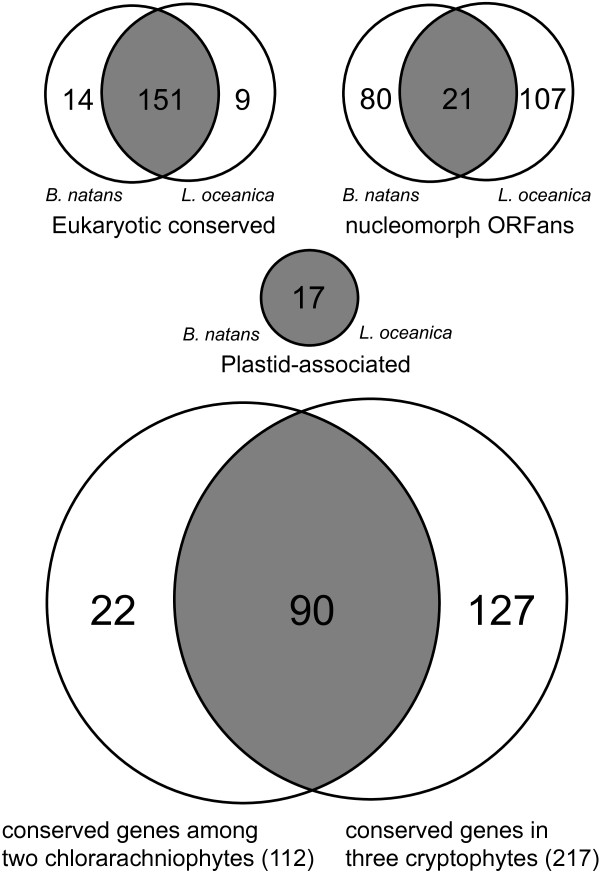
**Comparison of nucleomorph gene content within and between chlorarachniophyte and cryptophyte algae.** The Venn diagrams show the number of shared and / or unique genes in three categories: eukaryotic conserved (upper left), nucleomorph ORFans (upper right), and plastid-associated genes in the two chlorarachniophyte nucleomorph genomes (middle center), and core nucleomorph genes (excluding spliceosomal machinery genes and plastid-associated genes) in chlorarachniophytes and cryptophytes (bottom).

Compared to the non-redundant gene set in the *B. natans* nucleomorph genome, *L. oceanica* retains similar sets of eukaryotic conserved genes (151 of 160 *L. oceanica* protein genes in these categories are present in *B. natans*) (Figure 4, Additional files 1 and 3). 14 of these genes (rpl14A, rpl24, rpl30, rpoF, rps24-like, rpoL, rad25, dip2, rhel1, pcna, rfc4, msl1, snrpE-1, and ub2) are missing in *L. oceanica* but are present in the *B. natans* nucleomorph genome, and vice versa for nine *L. oceanica* genes (rpl15, rpl21A, nop56-like, cc1-like, psf2, ruvB2-like, tbl3, KH-domain and BRCA1) (Additional file 1). Despite missing several protein genes, the absence of whole suites of proteins predicted to function together in complexes was not observed in *L. oceanica*.

In stark contrast to the eukaryotic conserved genes with predicted functions, only 16.4-20.1% of nORFans were obviously shared between *L. oceanica* (21/128) and *B. natans* (21/101) (Figure 4). Interestingly, a similar tendency was observed when considering nucleomorph gene sets among three cryptophytes [20]. In this case, 93.0-99.6% of eukaryotic conserved ORFs (excluding spliceosome machinery genes) were shared with one another in *G. theta*, *H. andersenii* and *C. paramecium*, and only 12.2-23.2% for nORFans (Figure 4). The nORFans of chlorarachniophytes and cryptophytes have been proposed to represent particularly fast-evolving genes whose sequences have diverged to the point where their homologs in related nucleomorph genomes (and other nuclear genomes) can no longer be detected [9, 19–21]. Additionally, the *L. oceanica* and *B. natans* genomes encode the exact same set of 17 plastid-associated proteins. This parallels the cryptophyte genomes sequenced thus far (excluding the non-photosynthetic cryptophyte, *C. paramecium*), which share a set of 31 plastid-associated protein genes [9, 18–21].

The convergent evolution of nucleomorph genomes in cryptophytes and chlorarachniophytes is apparent from the perspective of G + C content, chromosome structure (in particular the rDNA-containing chromosome ends), and the presence of three linear chromosomes. Tanifuji *et al.*[20] extended this to the level of gene content, demonstrating that the majority of conserved eukaryotic genes (81.7%) in the *B. natans* nucleomorph genome were contained in the core set of cryptophyte nucleomorph genes, i.e., genes shared by all three photosynthetic cryptophytes (spliceosomal genes with discernable functions were excluded due to the fact that the cryptophyte *H. andersenii* lacks introns and thus spliceosomal machinery). This observation is bolstered by the present study. A ‘core’ set of 112 chlorarachniophyte nucleomorph genes was identified by comparison of the *L. oceanica* and *B. natans* gene sets (excluding plastid-associated and spliceosomal genes). 90 of these 112 genes (80.4%) were shared with at least one of the cryptophytes (which have a 217-gene core set) (Figure 4). There is now strong support for the idea that these two independently evolved nucleomorph genomes have undergone similar reductive pressures and converged upon similar gene sets.

The availability of a complete nuclear genome sequence for *B. natans* has allowed us to explore the fate of the nine ‘missing’ conserved eukaryotic genes in this organism (rpl15, rpl21A, nop56-like, cc1-like, psf2, ruvB2-like, tbl3, KH-domain and BRCA1). Curtis *et al.*[40] predicted 1,002 and 2,401 nucleus-encoded, periplastidal compartment (PPC)-targeted proteins in *B. natans* and the cryptophyte *G. theta*, respectively. Interestingly, in *G. theta* 16 of 17 genes missing from the nucleomorph genome but present in other cryptophyte nucleomorphs appear to have been lost without being replaced by a nucleus-encoded, PPC-targeted homolog (only the kinase-encoding gene kin(cdc) was potentially replaced by a host derived, PPC-targeted protein). As in cryptophytes, we were unable to identify obvious orthologs of the 9 genes missing from the *B. natans* nucleomorph genome that are present in the *L. oceanica* nucleomorph genome by searching the *B. natans* nuclear genome. Obvious replacements for the 19 protein genes missing from the *L. oceanica* nucleomorph genome were also not found in transcriptome data from this organism (a complete genome sequence is not available for analysis). That is, the genes do not appear to have undergone nucleomorph-to-host-nucleus gene transfer. One explanation is that the functions of these missing proteins have been taken over by functionally ambiguous nORFan proteins and/or nucleus-encoded, PPC-targeted hypothetical proteins. Determining whether this is the case will require much more comparative sequence data and a better understanding of the biochemical processes taking place in the PPC.

Several other factors also contribute to the larger nucleomorph genome size in *L. oceanica*. Unlike the *B. natans* genome where only an rps8 gene and sub-telomeric regions are multicopy, there are many multicopy regions in the *L. oceanica* nucleomorph genome. In addition to the sub-telomeric regions and tandem repeats containing the ClpC and tfIIa-gamma genes on chromosome I, this includes 15 internally duplicated regions (Figure 1) that result in numerous multicopy genes (e.g., eif4A, rpl10A, rpl23, rpl27, rps9, orf264, orf150, orf328, orf363 (2 copies each) and gsp2 (3 copies)). These genes contribute to the increase in the number of total protein genes in the *L. oceanica* genome (352, excluding ORFs in sub-telomeric regions), resulting in 69 more genes than in *B. natans* (283). Another contributing factor is a slightly larger average intergenic spacer size in *L. oceanica* relative to *B. natans* (Table 1). This mirrors the situation seen in the ‘large’ nucleomorph genome of the cryptophyte *C. mesostigmatica.* All things considered, a similar set of structural differences can explain the observed variation in nucleomorph genome size from species to species in both chlorarachniophytes and cryptophytes, despite their independent origins.

### Origins and evolution of chlorarachniophyte nucleomorph introns

A striking difference between chlorarachniophyte and cryptophyte nucleomorph genomes is the number of spliceosomal introns. Gilson *et al.*[17] found 852 pigmy introns from the *B. natans* nucleomorph genome, whereas cryptophyte nucleomorph genomes contain only 0–25 spliceosomal introns [17–21]. In this study we identified 1,011 *L. oceanica* nucleomorph introns using Illumina transcriptome data (RNA-Seq). For the *B. natans* nucleomorph, 115 new splice sites were also identified and corrected using Illumina RNA-Seq data. With these adjustments, the total number of spliceosomal introns in the *B. natans* nucleomorph genome has increased from 852 (the original estimate [17]) to 951. As in *B. natans*, the *L. oceanica* introns have canonical GT-AG intron boundaries, with the exception of three introns (two GC-AG and one GA-AG). Most of these introns are 18–23 bp in size. One intron, within an RNA helicase gene (prp43-2), was found to be 32 nt, which is the largest nucleomorph intron known (the previous largest was a 27 nt intron found in the rpb6 gene of the *Gymnochlora stellata* nucleomorph genome [27]).

**Figure 5:**
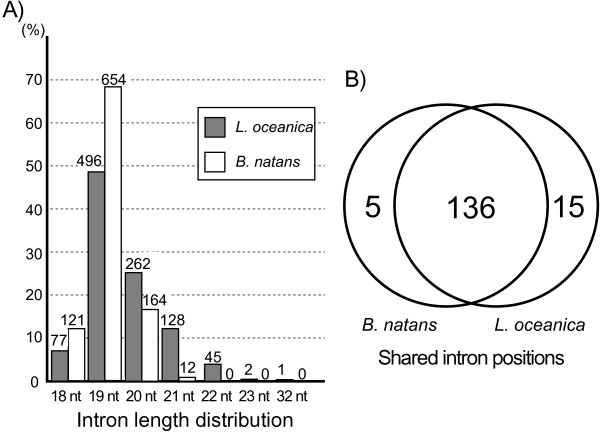
**Introns in the** ***Lotharella oceanica*** **and** ***Bigelowiella natans*** **nucleomorph genomes** ***.*** **A)** Intron size distribution for *L. oceanica* (left) and *B. natans* (right). The numbers above each bar show the actual numbers of introns in each size category. **B)** Intron comparison of two chlorarachniophyte nucleomorph genomes. The Venn diagram shows the number of shared and/ or unique comparable spliceosomal introns in the two genomes. Comparable spliceosomal introns were selected using the criteria proposed by Roy and Penny [30].

The size distribution of introns in the *L. oceanica* and *B. natans* genomes is shown in Figure 5. The pattern of intron abundance versus size in *L. oceanica* is similar to that in *B. natans* in that 19 nt-long introns are most abundant (496 in *L. oceanica* and 654 in *B. natans*), although the proportion of 19 nt introns in *L. oceanica* (49.1%) is smaller than in *B. natans* (68.8%). In *L. oceanica*, the size distribution is shifted towards longer introns (the proportions of introns <20 nt (18, 19 nt) are 56.7% and 81.5%, whereas the proportion of >20 nt introns are 43.3% and 18.5% in *L. oceanica* and *B. natans*, respectively) (Figure 5A). A previous comparison of introns in 54 homologous genes in the *B. natans* and *G. stellata* nucleomorph genomes [27] showed that the *G. stellata* genome also possesses a higher proportion of 19 nt-introns (78.3%) than *B. natans* (75.0%). The distribution pattern of intron size was somewhat shifted to longer intron size in *G. stellata* (20.3% and 13.3% of 20–24 nt intron in *G.stellata* and *B. natans*). The fact that the ~385 kbp *G. stellata* nucleomorph genome is estimated to be ~12 kbp larger than *B. natans* and tends to have longer introns than *B. natans* is consistent with our *L. oceanica* data in showing a positive correlation between nucleomorph genome size and intron length.

In terms of phase and base composition, the *L. oceanica* and *B. natans* nucleomorph introns are similar. Specifically, there is an abundance of phase 0 introns at similar proportions (58.3% in *L. oceanica* and 59.4% in *B. natans*), a bias towards A residues at position -2, and high A + T content in the intron sequences themselves (Additional file 4). The latter two features are also seen in *G. stellata* nucleomorph introns [27].

625 and 579 introns were found in 146 homologous nucleomorph genes shared between *L. oceanica* and *B. natans*. Of these introns, 151 *L. oceanica* and 141 *B. natans* introns were comparable under the criteria used by Roy and Penny [30] (see Methods). In terms of intron position, 136 introns were shared between these two species (Figure 5B). In order to gain insight into patterns of intron gain and loss during reductive evolution, 20 introns not shared between *L. oceanica* and *B. natans* were compared with those of two well-studied species, the plant *Arabidopsis thaliana* and the green alga *Chlamydomonas reinhardtii*. As shown in Additional file 5, one intron in rpc2 was absent only in *L. oceanica* and two introns (in the rpb10 and rpl44 genes) were absent only in *B. natans*. In these cases we can safely infer intron loss in *L. oceanica* and *B. natans*. Based on the fact that the intron density in the *B. natans* nucleomorph genome is similar to that of *C. reinhardtii* and *A. thaliana*, Slamovits and Keeling [27] suggested that nucleomorph intron loss is rare. Our comparison of *B. natans* and *L. oceanica* shows a high similarity in the number of introns in homologous genes, and is consistent with this notion.

### Phylogenetic analyses

A fundamental question in the evolution of chlorarachniophyte algae is the nature of the green algal endosymbiont that gave rise to the plastid and nucleomorph. Previous phylogenetic trees inferred from concatenated plastid-encoded proteins suggested that the chlorarachniophyte endosymbiont was related to the so-called TUC group of green algae, consisting of trebouxiophytes, ulvophytes, and chlorophytes, within the core chlorophytes [12, 41–44]. However, the precise position of the chlorarachniophyte plastid and nucleomorph within the TUC group was unclear. We attempted to address this issue by assembling and analyzing a large data set including both nucleomorph and plastid proteins.

**Figure 6:**
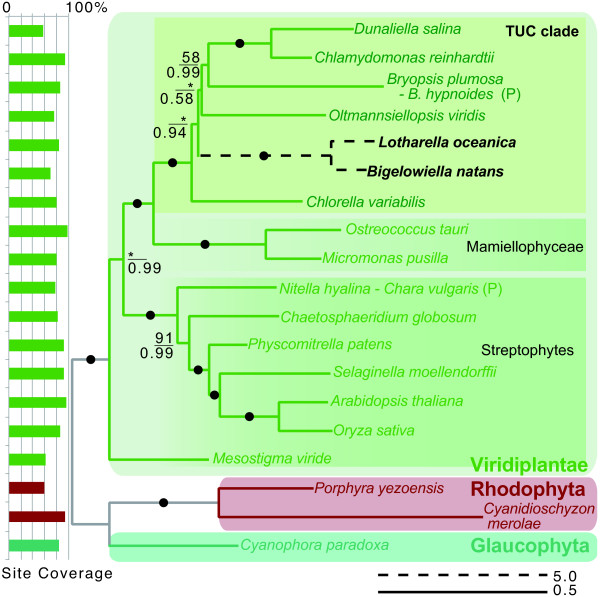
**Phylogenetic tree inferred from a concatenated set of 99 proteins (52 nucleomorph-/nucleus-encoded proteins and 47 plastid proteins) using PhyloBayes with the CAT + GTR + gamma model (4 rate categories).** Support values below the lines indicate Bayesian posterior probabilities, while the upper numbers are bootstrap support values based on RAxML. Black circles indicate branches supported with 100% bootstrap values and posterior probabilities of 1.0. Nodes where support values are less than 50% are shown with an asterisk (*). Scale bars shown by solid and broken lines indicate inferred number of amino acid substitutions per site. The fraction of amino acid sites present in the data matrix (site coverage) is shown on the left.

The taxon and data resources used in these analyses are shown in Additional file 6. Three separate supermatrices were constructed: (i) a 52-protein dataset containing nucleomorph and nuclear proteins (12,854 amino acid (AA) sites), (ii) a 47 plastid protein set (10,026 AA sites in total) and (iii) a 99-protein combined set (25,688 AA sites in total) (see Methods). Maximum likelihood trees inferred using the RAxML method [45] are shown in Additional file 7. As expected, *L. oceanica* and *B. natans* grouped within the Viridiplantae clade. The plastid protein tree and combined dataset tree place the two chlorarachniophyte species as monophyletic with the TUC clade with 100% bootstrap support. The relationship within the TUC clade was, however, unclear. In a Bayesian analysis using the Phylobayes method [46], *L. oceanica* and *B. natans* grouped with the TUC group and the Mamiellophyceae with maximum support (1.0 and 100%; Figure 6). Together, these analyses are consistent with the hypothesis that the endosymbiont that gave rise to the chlorarachniophyte nucleomorph and plastid was more closely related to members of the TUC group than the other green algal members. However, the branching pattern within the TUC clade, including the chlorarachniophytes, was not resolved by ML analysis (Figure 6 and Additional file 7c). Therefore, although the analyses presented herein use the most gene-rich datasets thus far assembled to address the question of the origin of the chlorarachniophyte endosymbiont, they still lack sufficient taxon sampling and the resolution needed to elucidate the relationship between chlorarachniophytes and the members of the TUC clade. *L. oceanica* and *B. natans* are clearly the longest branches in our trees, with the exception of the non-photosynthetic euglenoid, *Euglena longa,* in the plastid tree. These long branch lengths suggest that substitution rates in the nucleomorph and plastid genomes have accelerated after secondary endosymbiosis. Such long branches are well known as the cause of phylogenetic artifacts.

## Conclusions

We have sequenced the nucleomorph and plastid genomes of the chlorarachniophyte *L. oceanica*. The *L. oceanica* nucleomorph genome is the largest sequenced so far within the chlorarachniophytes, ~240 kbp larger than that of *B. natans*. Large, nearly identical sub-telomeric repeats on each of the six chromosome ends are the main contributors to its increased size, an apparent consequence of frequent homologous recombination at the chromosome ends that serve to expand and contract these regions in different species. Internally duplicated regions have also contributed to the secondary expansion of the *L. oceanica* nucleomorph genome. The *L. oceanica* nucleomorph genome contains 610 protein genes (348 non-redundant). 94.3% (151/160) of the eukaryotic conserved genes (i.e., ‘housekeeping’ genes) and all the plastid-associated genes were shared between *L. oceanica* and *B. natans*. Intron sizes and positions were also conserved. The presence of an overlap between the core gene sets in chlorarachniophyte and cryptophyte nucleomorph genomes speaks to convergent patterns of gene retention in these independently evolved nucleomorphs. In contrast to its dynamically evolving nucleomorph genome, the *L. oceanica* plastid genome is highly similar to the *B. natans* plastid genome in terms of genome size, gene content and gene synteny. Phylogenetic trees inferred from large datasets consisting of nucleomorph-/nucleus- and plastid-encoded proteins support the idea that the endosymbiont that gave rise to the chlorarachniophyte plastid and nucleomorph was a relative of the TUC group of green algae. Further phylogenetic resolution will require sequence data from more diverse members of the chlorarachniophytes, which will also improve our understanding of the tempo and mode of nucleomorph and plastid genome evolution in these enigmatic unicellular algae.

## Methods

### Cell Culturing, DNA extraction, and genome sequencing

*Lotharella oceanica* (CCMP622) cells were cultured in K media under 12:12 Light/Dark conditions at room temperature. Two-month old cells were then collected by centrifugation at 2,300 rpm for 15 min at 4°C. DNA extraction was done using a standard SDS-phenol/chloroform extraction method. 6 mg of total cellular DNA was separated by Hoechst dye (No 33258, Sigma-Aldrich, St. Louis, MO, USA)-cesium chloride density gradient centrifugation at 35,000 rpm for 65 hrs at 4°C as described previously [19–21]. Discrete bands were purified and gradient centrifugation was repeated twice using the same conditions to increase purity. The genomic identities of the resulting four DNA fractions were determined by semi-quantitative PCR using four primer sets which specifically amplified nuclear (actin), nucleomorph (18S rDNA), plastid (rbcL), and mitochondrial (cox1) genes. A DNA fraction (8.4 μg) found to be enriched with nucleomorph and plastid DNA was sent to the Genome Quebec Innovation Center (Montreal, Quebec) for TruSeq library construction and Illumina HiSeq2000 sequencing.

### RNA extraction and transcriptome sequencing

Two-month old cell cultures were transferred to new culture flasks with a half volume of new K media three days before RNA extraction. Cells were harvested at 2,300 rpm for 15 min at 4°C and RNA was extracted using TRizol (Invitrogen). Extracted total RNA was digested with DNase I at 37°C for 15 min and purified using the RNeasy^®^ Mini Kit (Qiagen, Toronto, ON, Canada). 5 μg of total RNA was used for sequencing on the Illumina HiSeq2000 platform and reads were assembled into contigs at the National Center for Genome Resources (Santa Fe, NM, USA) within the context of the Marine Microbial Eukaryote Transcriptome Project (http://marinemicroeukaryotes.org/). The data was deposited in the CAMERA portal [47] under the project ID MMETSP0040.

### Nucleomorph and plastid genome assembly

424,477,680 paired-end reads (100 base read lengths) were trimmed to 95 bases where the mean base quality score was >32 (Illumina 1.8+, phred + 33) using the FASTX-Tool kit (Ver.0.0.13) (http://hannonlab.cshl.edu/fastx_toolkit/). The sequence assembly was done using PASHA (ver. 1.0.3) [48] with a kmer size of 31. The first assembly generated ~1,800 scaffolds >5 kbp. The three largest scaffolds (93,892 bp, 89,349 bp, and 46,146 bp) were identified as nucleomorph with a depth of coverage of ~7,000× and with a G + C content of ~20% (The mapping strategy for calculating coverage is described in the next section). No scaffolds of plastid origin were identified in the first assembly. The reads used to construct scaffolds in the first assembly were filtered out, leaving 246 million reads. In our experience, scaffolds with extremely high levels of coverage tend to be ignored during assembly. Therefore, only 1,700,000 reads were used for the second-round assembly of the plastid genome. Four scaffolds were generated, all of which were found to be plastid in origin (12,337 bp, 9,456 bp, 28,109 bp, and 6,832 bp) with a depth of coverage ~1,600×. Two plastid-derived contigs from the transcriptome data (6,735 bp and 7,150 bp) were also used for subsequent manual assembly.

For the nucleomorph genome, paired-reads corresponding to the plastid genome and/or low coverage scaffolds (<1,000×)) in the first assembly were discarded, after which ~30 million paired-end reads remained. Only 2 of the 30 million paired-end reads were used for the third assembly in order to avoid problems associated with high coverage depths. 40 scaffolds (177 contigs) were generated with ~200× coverage. All scaffolds with a size of >4.5 kbp (11 scaffolds, 370,802 bp in total) were clearly nucleomorph in origin.

To fill gaps between/within scaffolds and contigs, 51 and 17 primers were designed for the nucleomorph and plastid genomes, respectively. Primers were also designed against five loci of the multicopy hsp70 gene, including a frame-shifted gene and one with an internal stop codon, to verify sequences. PCR was done using Takara exTaq and PCR products were cloned into pGEM T easy vector (Promega). The cloned samples were sent for Sanger sequencing at the Genwiz sequencing facility. To verify the structure of chromosomal repeats, long range PCR was performed using Takara LA Taq with primers that were designed against unique internal regions of the chromosomes and the chromosomal repeats themselves.

### Genome mapping, intron detection, and intron comparison

21,095,918 RNA-Seq reads (100 base-pairs long) were trimmed to 85 bases where the mean base quality score was above 24 (Illumina 1.5+, phred + 64) using the FASTX-Tool kit (Ver.0.0.13). All reads were mapped to the three complete nucleomorph chromosomes as a reference using BWA (Burrows-Wheeler aligner, ver. 0.6.2) [49]. For transcriptome analyses, the base coverage at each nucleotide position was calculated using the mpileup function in Samtools (ver. 0.1.18 ) [50]. The depth of coverage for each gene was calculated by summing up the coverage at specific gene coordinates and normalizing the output for gene length using in-house perl scripts, as in Tanifuji et al. [37]. Intron positions were detected manually from the BWA mapping results and visualized using the Integrative Genome Viewer (IGV ver.2.0) [51]. RNA-Seq transcriptome data for *B. natans* (MMETSP0045) were also mapped to the nucleomorph genome of this organism (accession numbers NC_010004.1, NC_010005.1 and NC_010006.1), with the goal of verifying intron predictions based on Sanger EST sequencing by Gilson *et al.*[17]. For comparison of intron positions between *L. oceanica* and *B. natans*, 145 homologous genes were individually aligned using clustalW (ver. 2.1) [52] and intron positions within high-quality alignment regions were compared (a minimum of 15 gap-free amino acid residues on either side of the intron-containing codon with >50% identity were retained for downstream analysis, as in Roy and Penny [30]). In order to verify intron presence/absence in *A. thaliana* and *C. reinhardtii* for 20 intron sites not shared between *B. natans* and *L. oceanica*, nuclear genome sequences and annotations were obtained from the public database of the National Center for Biotechnology Information (NCBI) for *A. thaliana* (NC_003070-NC003071, NC_003074-NC003076) and The Joint Genome Institute *Chlamydomonas* v4 portal for *C. reinhardtii*[28]. Orthologous genes in *A. thaliana* and *C. reinhardtii* were identified by BlastP using nucleomorph protein sequences from *B. natans* and *L. oceanica* as queries. The protein sequences from all four species were aligned using clustalW (ver. 2.1) [52]; regions with >40% amino acid sequence identity were compared; introns were retained for further analysis if the criteria of Roy and Penny [30] were met. The graphs of sequence conservation and base frequencies of introns and their flanking regions were generated using WebLogo 3 [53]. Gene expression analysis for *B. natans* was carried out as described previously [37].

### Gene annotation

After the identification of introns in the *L. oceanica* nucleomorph genome, ORFs greater than 50 amino acids were identified using Artemis (Ver.13.0) [54]; ORFs in the plastid genome were identified similarly. Blastx and blastp searches were done for all ORFs against the *B. natans* nucleomorph genome (Accession No. NC_010004.1, NC_010005.1 and NC_010006.1) and plastid genome sequence data (Accession No. NC_008408.1) with an e-value cut-off of 0.01. The non-redundant (nr) database was also searched with a cut-off e-value of 0.001. Each predicted gene was functionally categorized according to Tanifuji *et al.*[20]. Transfer RNA prediction was done by tRNAscan-SE ver.1.2.1 [55] using a ‘Eukaryotic’ source for the nucleomorph genome and a ‘Mito/Chloroplast’ source for the plastid genome. Ribosomal RNA operons were identified using published data (Accession No. HQ009889).

### Average protein lengths and intergenic spacer sizes

The mean length of proteins shared between *L. oceanica* and *B. natans* (excluding nORFans) was calculated using 166 proteins. The mean length for all proteins was calculated using 610 and 284 proteins in *L. oceanica* and *B. natans*, respectively, and the average intergenic spacer sizes were calculated using 633 (*L. oceanica* and 305 (*B. natans*) intergenic spacers. T-tests and paired T-tests were performed using R statistical software (Ver. 2.14.1) [56].

### Phylogenetic analysis of hsp70

A dataset of 34 hsp70 amino acid sequences (including mitochondrial, plastid, cytoplasmic, and ER isoforms) from *Cyanidioschyzon merolae*, *Thalassiosira pseudonana*, *Ostreococcus tauri*, *Synecocystis* sp. Strain CC6803, *C. reinhardtii*, and *A. thaliana* was assembled according to Renner and Waters [57]. Including the sequences of three hsp70 isoforms encoded in the *L. oceanica* nucleomorph genome and one from *B. natans*, all 38 amino acid sequences were aligned using MAFFT version 7 [58]. Alignments were then trimmed automatically using TrimAl v1.2 [59] with a gap threshold of 0.8 and a similarity threshold of 0.001. A phylogenetic tree was constructed using RAxML ver. 7.0.3 [45] with the LG substitution matrix + Gamma + Invar (4 site rate categories). Bootstrap values were calculated with the rapid bootstrap method with 1,000 replicates.

### Phylogenomic dataset construction

77 nucleomorph and 47 plastid proteins shared among chlorarachniophytes and cryptophytes were selected from the *L. oceanica* genomic data and used to construct a phylogenomic dataset (multicopy genes and genes/proteins showing low similarity to homologs in other organisms were excluded). First, the protein sequences for each *L. oceanica* protein were used to identify homologs using blastp against OrthoMCL v.5 (http://www.orthomcl.org) with an e-value cutoff of 1e-5. The OrthoMCL ID of the top hit for each input protein was then collected and compared against all other input protein OrthoMCL IDs. If an OrthoMCL ID was present more than once, these genes were removed from consideration, as it may be the result of paralogy. The remaining OrthoMCL IDs were then used to create a reference ortholog dataset by collecting the corresponding orthologs (based on OrthoMCL ID) from *Chlamydomonas reinhardtii* in the OrthoMCL database. In cases where a *C. reinhardtii* ortholog was not present in the OrthoMCL database, the corresponding ortholog from *Arabidopsis thaliana, Cyanidioschyzon merolae, Ostreococcus lucimarinus,* or *Volvox carteri* f. *nagariensis* was collected (see Additional file 6).

Various organismal genomic/transcriptomic data and plastid data collected from publically available sources were used as input for an in-house pipeline, described below, for the creation of single protein datasets and, subsequently, the phylogenomic data matrix. The organismal data were individually screened for orthologs using either blastp or tblastn, depending on the data type, with the above reference ortholog sequences used as queries in Blastmonkey from the Barrel-o-Monkeys toolkit (http://rogerlab.biochem.dal.ca). If the sequence dataset was nucleotides, then the tblastn hits were translated to amino acid residues. Blastp was then used to screen these putative orthologs against the OrthoMCL database, and the output for each gene from each organism was compared against a dictionary of orthologous OrthoMCL IDs. Those putative orthologs that did not match orthologous IDs were designated as paralogs and removed. The remaining orthologs from each organism were combined and aligned using MAFFT-LINSI [58]. Ambiguously aligned positions were identified and removed using Block Mapping and Gathering with Entropy (BMGE) [60]. For each individual protein alignment, maximum-likelihood (ML) trees were inferred in RAxML v7.2.6 [46] using the LG + gamma distribution with four rate categories, with 10 ML tree searches and 100 ML bootstrap (MLBS) replicates. To test for undetected paralogy or contaminants, we constructed a consensus tree (ConTree) representing phylogenetic groupings of well-established eukaryotic clades [61–65]. The resulting individual nucleomorph/nuclear protein trees that placed taxa in conflicting positions relative to the ConTree with more than 70% maximum likelihood bootstrap support (MLBS), with a zero-branch length, or with extremely long branches were checked manually. These sequences were further scrutinized for hidden paralogy and contamination issues through reciprocal blastp against *Oryza sativa* orthologs in NCBI. All problematic sequences identified using these methods were removed from the dataset. The resulting protein alignments were then re-trimmed for ambiguously aligned positions using BMGE and concatenated into three separate supermatrices; a nuclear/nucleomorph dataset (52 proteins: 12,854 amino acid (AA) sites), a plastid dataset (47 proteins: 10,026 AA sites), and a combined nuclear-nucleomorph/plastid dataset (99 proteins: 25,688 AA sites), using a in-house script employing alvert.py from the Barrel-o-Monkey’s toolkit. For the combined nuclear/plastid dataset, taxon sampling was reduced to focus specifically on plants/algae and chlorarachniophyte taxa whose plastid genomes were available along with a sufficient amount of nuclear/nucleomorph data. In this dataset two chimeras were constructed using nuclear and plastid data from different but closely related taxa, namely *Bryopsis plumosa* (nuclear) × *B. hypnoides* (plastid) and *Nitella hyalina* (nuclear) × *Chara vulgaris* (plastid). Data sources, details on gene sampling, and information on missing data are in Additional file 6.

### Phylogenomic analyses

ML trees for each dataset were estimated from 60 independent searches using RAxML under an LG + gamma (four rate categories) and an empirical amino acid frequencies model, selected by the Akaike information criterion. Topological support was assessed using 1,000 RAxML bootstrap replicates (Additional file 7). For the combined nucleomorph-nucleus/plastid dataset, Bayesian inferences (BI) were made in PhyloBayes-MPI [46] under the CAT-GTR + gamma (four rate categories) model, and two independent Markov chain Monte Carlo chains were run for 8,000 generations sampling every two generations. For PhyloBayes analyses, constant sites were removed to decrease computational time. Convergence was achieved for the chains at 400 generations, with the largest discrepancy in posterior probabilities (maxdiff) <0.012 and the effective size of continuous model parameters were in the range of acceptable values (>50). Posterior probabilities of post-burn-in bipartitions (2 chains, 3,600 trees, sampling every 10 trees) were mapped on to the consensus BI topology (Figure 6). The MLBS values were mapped onto this tree.

### Availability of supporting data

Nucleomorph and plastid genome sequences and annotations were deposited in GenBank under the following accession numbers; nucleomorph chromosomes 1–3 (CP006627-CP006629), plastid genome (KF438023). The RNA-Seq data is available in the CAMERA portal (http://camera.calit2.net/) under the project ID MMETSP0040. Supporting protein alignment datasets used in this work are available from the LabArchives (http://www.labarchives.com/) repository under doi:10.6070/H4BV7DJZ.

### Ethics

Ethics approval was not required for the research described in this manuscript.

## Electronic supplementary material

Additional file 1: **Nucleomorph Gene Content List of** ***L. oceanica*** **and** ***B. natans.*** (PDF 98 KB)

Additional file 2: **RaxML tree inferred from hsp70 proteins.** (PDF 325 KB)

Additional file 3: **Venn Diagram of gene content between** ***L. oceanica*** **and** ***B. natans.*** (PDF 308 KB)

Additional file 4: **Base composition of introns and their flanking regions.** (PDF 512 KB)

Additional file 5: **Intron comparison between hlorarachniophytes and green algae.** (PDF 271 KB)

Additional file 6: **Taxon list and resource for phylogenetic analysis.** (PDF 41 KB)

Additional file 7: **RAxML trees inferred from different protein sets.** (PDF 563 KB)
