# Supplementary material for: Nucleomorph and plastid genome sequences of the chlorarachniophyte Lotharella oceanica: convergent reductive evolution and frequent recombination in nucleomorph-bearing algae
Source: BMC Genomics. 2014 May 15;15(1):374. doi: 10.1186/1471-2164-15-374 (PMC4035089; doi:10.1186/1471-2164-15-374)
Supplement: Supplementary file 1 — Additional file 1: Nucleomorph Gene Content List of L. oceanica and B. natans. (PDF 98 KB) [file 12864_2014_6068_MOESM1_ESM.pdf]

Additional file1; Nucleomorph Gene Content List of *L. oceanica* and *B. natans*
Blue=missing in *L. oceanica* nm*,* Red=missing in *B. natans* nm

Translation

dbp1, ef2, eif1A, eif2G, eif4A, eif6, rla0, rpl1, rpl10, rpl10A, rpl11B, rpl13A, rpl14A, rpl15, rpl17, rpl18A, rpl19, rpl2, rpl21A, rpl23, rpl24, rpl27, rpl27A, rpl3, rpl30, rpl32, rpl34, rpl37A, rpl44, rpl5, rpl7, rpl7A-1, rpl7A-2, rpl8, rpl9, rps10B, rps11, rps12, rps13, rps14, rps15, rps15A, rps16, rps17E, rps18, rps2, rps23, rps24-like, rps26, rps27, rps27A, rps28, rps3, rps30, rps3A, rps4, rps5, rps6, rps7, rps8, rps9, rsp4, sys1

Transcription

myb1, rpa1, rpa2, rpa5, rpabc5, rpabc6, rpb1, rpb10, rpb3, rpb11, rpb2, rpb8, rpc1, rpc10, rpc2, rpoF, rpoL, tfIIA-gamma, tfIIB, tfIID, tfIIIB

Protein Folding and Degradation

hsp70, hsp90, hub1, tcpB, tcpD, tcpE, tcpG, tcpH, tcpT

Mitosis

cdc5

DNA Metabolism and Cell Cycle

cdc48, h3, h4, mak16, mcm2, mcm4, pcna, psf2, rad25, rad51, rfc4, ruvB2-like

Spliceosomal Machinery

cc1-like, cdc28, clf1, cwc22, cwc24, dib1, duf572, G10, mcf1.25, msl1, phf5-like, prl1, prp14, prp16, prp17, prp19, prp22, prp38, prp43-1, prp43-2, prp45, prp6, prp8, sap62, sf3a1, sf3a3, sf3b,sf3b4, sf3b5, sf3bA, sfb1, snrpE-1, snrpE-2, snrpB, snrpD, snrpD2, snrpD3, snrpG, snrpF, u2AF, U5snRNP-116kDa, U5snRNA-200kDa, ub2

RNA Metabolism

ATP/GTP-bp, dip2,fcf1, gsp2, imp4, mago, mis3, ngp1, ngp2, nip7, nop1, nop2, nop56, nop56-like, ppci, rhel1,rnabp1, rnabp2, sbp1, ysh1

Plastid-Associated

clpC, clpP1, clpP2, clpP3, clpP4, clpP5, clpP6, cpn60, dnaK, murL, rpoD, secY, sufB, tatC, tic20, toc75, ycf16

Miscellaneous

mrp1, tbl3

ORF with Motifs

KH-domain, conserved ATPase, BRCA1

rRNAs, and tRNAs(anticodon)

5.8SrRNA, 18SrRNA, 28SrRNA
trnV(UAC), trnH(GUG), trnW(CCA), trnI(AAU), trnQ(CUG), trnA(UGC), trnD(GUC), trnS(GCU), trnC(GCA), trnV(AAC), trnM(CAU), trnE(UUC), trnL(UAG), trnI(UAU), trnY(GUA), trnF(GAA), trnN(GUU), trnL(CAA), trnV(GAC), trnT(AGU), trnK(CUU)

ORFs shared between *L. oceanica* and *B. natans*

orf363(BNATCHR197), orf328(BNATCHR1116), orf144(BNATCHR2118), orf158(BNATCHR359), orf200(BNATCHR290), orf136(BNATCHR1107), orf333(BNATCHR362), orf106(BNATCHR2116), orf796(BNATCHR2114), orf75(BNATCHR2101), orf103(BNATCHR297), orf175(BNATCHR2108), orf400(BNATCHR284), orf699(BNATCHR282), orf195(BNATCHR374), orf450(BNATCHR1110), orf310(BNATCHR1111), orf729(BNATCHR194), orf1420(BNATCHR184), orf616(BNATCHR113), orf326(BNATCHR2105)
