# Supplementary figures and images for: Nucleomorph and plastid genome sequences of the chlorarachniophyte Lotharella oceanica: convergent reductive evolution and frequent recombination in nucleomorph-bearing algae
Source: BMC Genomics. 2014 May 15;15(1):374. doi: 10.1186/1471-2164-15-374 (PMC4035089; doi:10.1186/1471-2164-15-374)

Additional file 2; RaxML tree inferred from hsp70 proteins

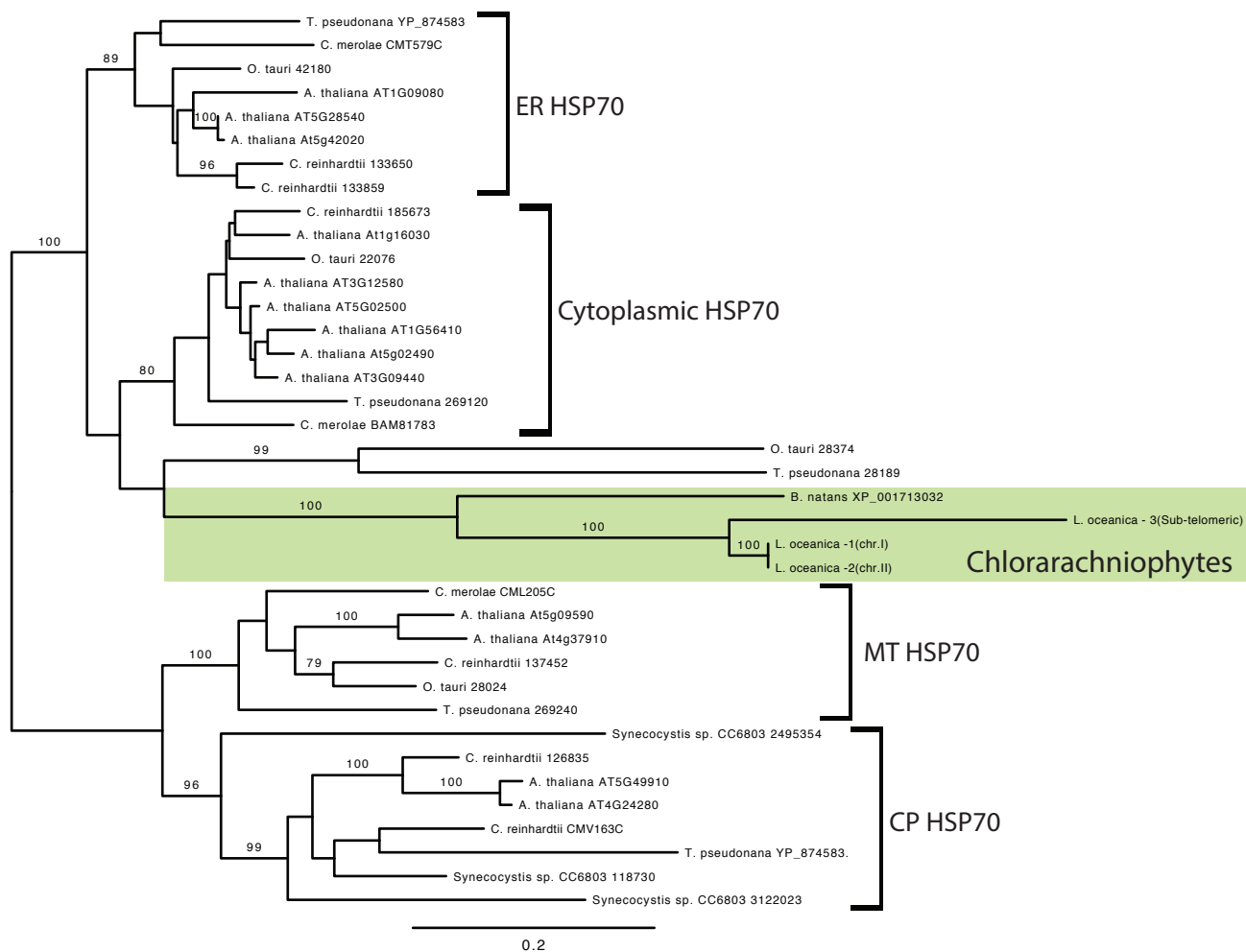

Supplement: Supplementary file 2 — Additional file 2: RaxML tree inferred from hsp70 proteins. (PDF 325 KB) [file 12864_2014_6068_MOESM2_ESM.pdf]
