# Supplementary material for: Nucleomorph and plastid genome sequences of the chlorarachniophyte Lotharella oceanica: convergent reductive evolution and frequent recombination in nucleomorph-bearing algae
Source: BMC Genomics. 2014 May 15;15(1):374. doi: 10.1186/1471-2164-15-374 (PMC4035089; doi:10.1186/1471-2164-15-374)
Supplement: Supplementary file 3 — Additional file 3: Venn Diagram of gene content between L. oceanica and B. natans. (PDF 308 KB) [file 12864_2014_6068_MOESM3_ESM.pdf]

Additional file 3; Venn Diagram of gene content between *L. oceanica* and *B. natans*

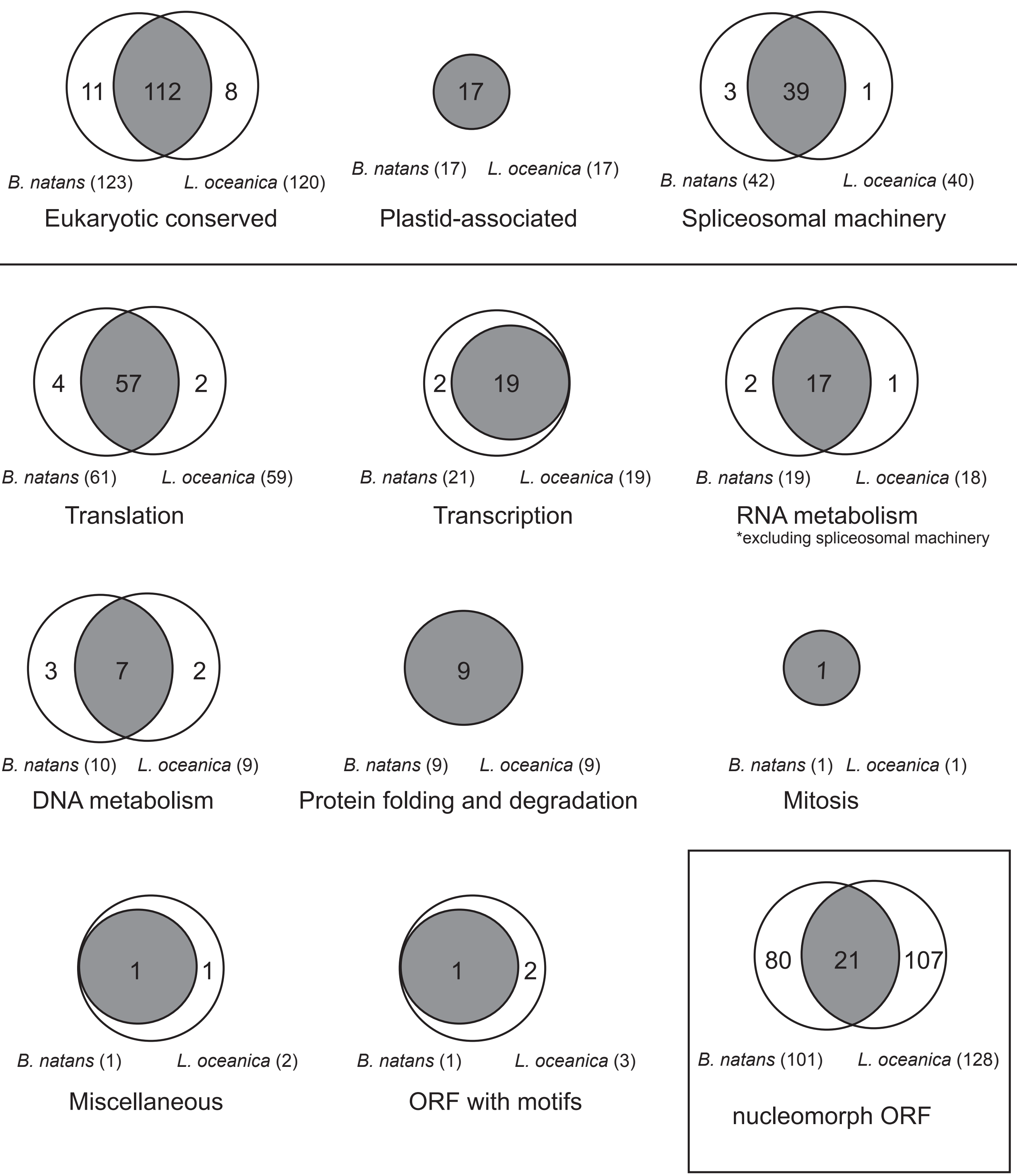

*The numbers are calculated based on non-redundant gene sets.

*Three genes are newly annotated from B.natans nm genome (rps24-like, rpoL and tfIII-brf)
