# Supplementary material for: Nucleomorph and plastid genome sequences of the chlorarachniophyte Lotharella oceanica: convergent reductive evolution and frequent recombination in nucleomorph-bearing algae
Source: BMC Genomics. 2014 May 15;15(1):374. doi: 10.1186/1471-2164-15-374 (PMC4035089; doi:10.1186/1471-2164-15-374)

Additional file 4; Base composition of intron and its flanking region.

a) 18nt intron

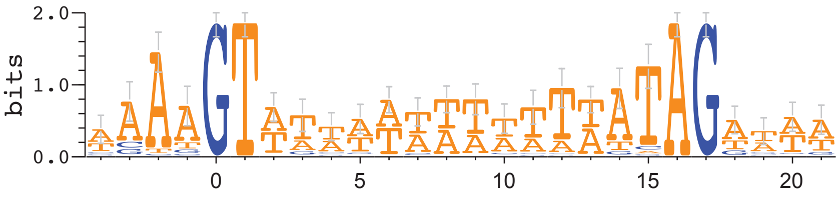

b) 19nt intron

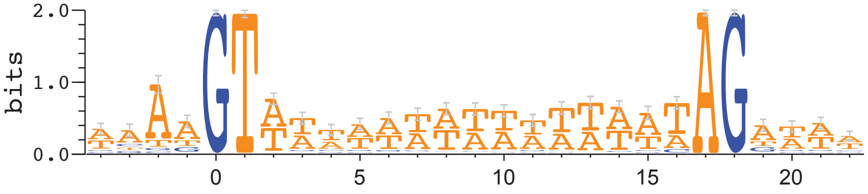

c) 20nt intron

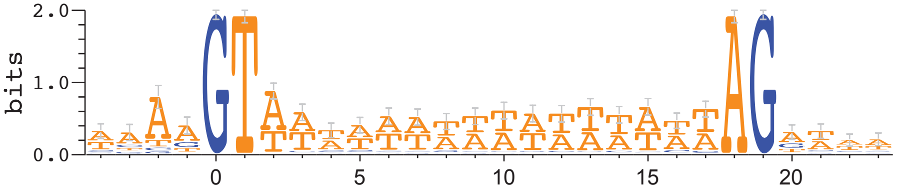

d) 21nt intron

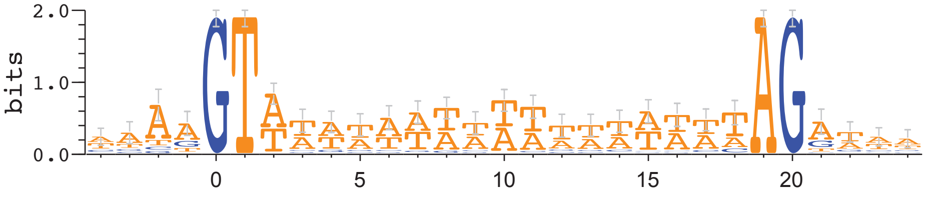

e) 22nt intron

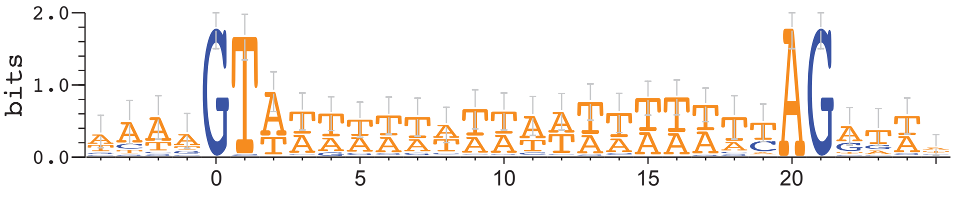

Supplement: Supplementary file 4 — Additional file 4: Base composition of introns and their flanking regions. (PDF 512 KB) [file 12864_2014_6068_MOESM4_ESM.pdf]
