# Supplementary material for: Nucleomorph and plastid genome sequences of the chlorarachniophyte Lotharella oceanica: convergent reductive evolution and frequent recombination in nucleomorph-bearing algae
Source: BMC Genomics. 2014 May 15;15(1):374. doi: 10.1186/1471-2164-15-374 (PMC4035089; doi:10.1186/1471-2164-15-374)
Supplement: Supplementary file 5 — Additional file 5: Intron comparison between hlorarachniophytes and green algae. (PDF 271 KB) [file 12864_2014_6068_MOESM5_ESM.pdf]

Additional file 5; Intron comparison between chlorarachniophyte and green algae

gene	*L. oceanica*	*B. natans*	*C. reinhardtii*	*A. thaliana*
myb1	-	+	incomparable	incomparable
rpb2	-	+	incomparable	incomparable
rpc2	-	+	+	+
rpl3	-	+	+	-
sf3a3	-	+	-	-
ef2	+	-	+	-
murL	+	-	incomparable	incomparable
myb1	+	-	-	-
phf5-like	+	-	+	-
prp17	+	-	incomparable	incomparable
rpa2	+	-	incomparable	incomparable
rpb1	+	-	incomparable	incomparable
rpb10	+	-	+	+
rpb2	+	-	-	+
rpb2	+	-	incomparable	incomparable
rpc2	+	-	incomparable	incomparable
rpc2	+	-	-	-
rpl44	+	-	+	+
rps11	+	-	incomparable	incomparable
sf3a3	+	-	-	-
