# Supplementary material for: Nucleomorph and plastid genome sequences of the chlorarachniophyte Lotharella oceanica: convergent reductive evolution and frequent recombination in nucleomorph-bearing algae
Source: BMC Genomics. 2014 May 15;15(1):374. doi: 10.1186/1471-2164-15-374 (PMC4035089; doi:10.1186/1471-2164-15-374)
Supplement: Supplementary file 6 — Additional file 6: Taxon list and resource for phylogenetic analysis. (PDF 41 KB) [file 12864_2014_6068_MOESM6_ESM.pdf]

Additional file 6; Taxon list and resource for phylogenetic analysis

RefDat

Taxon	OG5 Source ID or NCBI accession	OG5 ID	SwissProt Name
**NUCLEOMORPH**			
Chlamydomonas reinhardtii	24524	126923	EF2
Chlamydomonas reinhardtii	114706	126573	H4
Chlamydomonas reinhardtii	185673	126588	HSP70
Ostreococcus lucimarinus	XP_001419938	126623	HSP90
Chlamydomonas reinhardtii	141865	127127	IF1AX
Chlamydomonas reinhardtii	188065	127488	IF62
Chlamydomonas reinhardtii	127225	127912	IMP4
Chlamydomonas reinhardtii	206198	128004	MAK16
Chlamydomonas reinhardtii	38989	126796	NOP2
Chlamydomonas reinhardtii	38371	128467	NVL
Chlamydomonas reinhardtii	162845	126957	R13A1
Chlamydomonas reinhardtii	155455	127016	R27A3
Chlamydomonas reinhardtii	128522	127514	RANA1
Chlamydomonas reinhardtii	608	127927	RH34
Chlamydomonas reinhardtii	195585	127114	RL10A
Chlamydomonas reinhardtii	195587	126688	RL11
Chlamydomonas reinhardtii	76376	127033	RL15
Chlamydomonas reinhardtii	77517	126900	RL17
Chlamydomonas reinhardtii	126189	126874	RL3
Chlamydomonas reinhardtii	191758	126965	RL32
Chlamydomonas reinhardtii	126853	127166	RL37A
Chlamydomonas reinhardtii	195598	127050	RL4B
Chlamydomonas reinhardtii	183782	127090	RL5

Chlamydomonas reinhardtii	139831	126993	RL9
Chlamydomonas reinhardtii	176094	127924	RPA1
Chlamydomonas reinhardtii	113199	127929	RPA2
Chlamydomonas reinhardtii	151082	127455	RPAB1
Chlamydomonas reinhardtii	107215	127345	RPAB2
Chlamydomonas reinhardtii	127606	127691	RPAB5
Arabidopsis thaliana	NP_176261	127819	RPAC1
Chlamydomonas reinhardtii	111305	127622	RPB3A
Chlamydomonas reinhardtii	195602	126722	RPS2
Chlamydomonas reinhardtii	169450	127089	RS13
Chlamydomonas reinhardtii	195606	126930	RS16
Chlamydomonas reinhardtii	205854	127029	RS23
Chlamydomonas reinhardtii	46276	127138	RS26
Chlamydomonas reinhardtii	150644	127008	RS27
Chlamydomonas reinhardtii	144646	127046	RS28
Chlamydomonas reinhardtii	134051	126820	RS3
Chlamydomonas reinhardtii	132716	127269	RS30
Chlamydomonas reinhardtii	168484	126852	RS3A
Chlamydomonas reinhardtii	191776	127064	RS5
Chlamydomonas reinhardtii	104348	126964	RS6
Chlamydomonas reinhardtii	195593	126983	RS9
Chlamydomonas reinhardtii	126059	127014	RSSA
Chlamydomonas reinhardtii	105410	126871	SPB1
Chlamydomonas reinhardtii	184701	127730	TCPB
Chlamydomonas reinhardtii	107474	127832	TCPD
Chlamydomonas reinhardtii	131113	127343	TCPE
Chlamydomonas reinhardtii	116746	127782	TCPH
Chlamydomonas reinhardtii	55056	127923	TCPQ

Chlamydomonas reinhardtii	129972	127428	TCPZ

PLASTID

Chlamydomonas reinhardtii	195724	126818	ATP9
Chlamydomonas reinhardtii	76602	127165	ATPAM
Cyanidioschyzon merolae	CMH197C	127099	ATPB
Cyanidioschyzon merolae	CMV223C	171675	ATPF
Cyanidioschyzon merolae	CMV096C	128504	CYB6
Cyanidioschyzon merolae	CMV155C	157648	CYF
Chlamydomonas reinhardtii	98182	126954	EFTU
Cyanidioschyzon merolae	CMV097C	146609	PETD
Cyanidioschyzon merolae	CMV139C	196650	PETG
Cyanidioschyzon merolae	CMV135C	165873	PSAA
Cyanidioschyzon merolae	CMV136C	151231	PSAB
Cyanidioschyzon merolae	CMV059C	155541	PSAC
Cyanidioschyzon merolae	CMV202C	196653	PSAJ
Cyanidioschyzon merolae	CMV047C	151230	PSBA
Cyanidioschyzon merolae	CMV124C	157647	PSBB
Cyanidioschyzon merolae	CMV082C	172393	PSBC
Cyanidioschyzon merolae	CMV081C	172392	PSBD
Cyanidioschyzon merolae	CMV231C	172394	PSBE
Cyanidioschyzon merolae	CMV230C	196656	PSBF
Cyanidioschyzon merolae	CMV127C	165872	PSBH
Cyanidioschyzon merolae	CMV240C	196657	PSBI
Cyanidioschyzon merolae	CMV228C	181206	PSBJ
Cyanidioschyzon merolae	CMV140C	196651	PSBK
Cyanidioschyzon merolae	CMV229C	196655	PSBL
Cyanidioschyzon merolae	CMV126C	196648	PSBN
Cyanidioschyzon merolae	CMV125C	225209	PSBT

Cyanidioschyzon merolae	CMV013C	138568	RBL
Cyanidioschyzon merolae	CML196C	126688	RL11
Chlamydomonas reinhardtii	137303	126678	RL14
Chlamydomonas reinhardtii	131648	127517	RL16
Chlamydomonas reinhardtii	116036	126641	RL2
Chlamydomonas reinhardtii	101876	128492	RL20
Cyanidioschyzon merolae	CMP301C	128944	RM36
Chlamydomonas reinhardtii	169121	126637	RPC1
Cyanidioschyzon merolae	CMV185C	130460	RPOA
Cyanidioschyzon merolae	CMV216C	130697	RPOB
Volvox carteri f. *nagariensis*	XP2959920	136933	RPOC2
Chlamydomonas reinhardtii	118993	126681	RS11
Chlamydomonas reinhardtii	130212	127680	RS12
Chlamydomonas reinhardtii	24289	126687	RS15
Cyanidioschyzon merolae	CMP223C	128626	RS18
Cyanidioschyzon merolae	CMT330C	128046	RS2
Cyanidioschyzon merolae	CMN148C	126820	RS3
Cyanidioschyzon merolae	CMV009C	131566	RS4
Chlamydomonas reinhardtii	147018	127867	RS7
Cyanidioschyzon merolae	CMV177C	130342	RS8
Chlamydomonas reinhardtii	81150	127575	RT14

Taxon Abbreviation	Taxon Name	Source
**NUCLEAR**		
Acetacet	*Acetabularia acetabulum*	NCBI
Ampqueen	*Amphimedon queenslandica*	NCBI
AndaInca	*Andalucia incarcerata*	TBestDB
Arabthal	*Arabidopsis thaliana*	NCBI
Bigenata	*Bigelowiella natans*	JGI
Blashomi	*Blastocystis hominis*	NCBI
Bodosalt	*Bodo saltans*	Sanger
Branflor	*Branchiostoma floridae*	NCBI
Bryoplum	*Bryopsis plumosa*	Plant 1KP
Calltube	*Calliarthron tuberculosum*	http://dbdata.rutgers.edu/data/plantae/
Capsowcz	*Capsaspora owczarzaki*	NCBI
Chaeglob	Chaetosphaeridium globosum	NCBI
Chalrein	*Chlamydomonas reinhardtii*	NCBI
Chloatmo	*Chlorokybus atmophyticus* UTEX2591	NCBI
ChloNC64	*Chlorella* sp. NC64	JGI
Coprcine	*Coprinus cinereus*	Broad
Cryp2293	Cryptophyceae sp. CCMP2293	NCBI SRA
CyanMero	*Cyanidioschyzon merolae*	NCBI
Cyanpara	*Cyanophora paradoxa*	http://cyanophora.rutgers.edu/
DictDisc	*Dictyostelium discoideum*	DictyBase
Dunasali	*Dunaliella salina*	NCBI
Ectosili	*Ectocarpus siliculosus*	http://bioinformatics.psb.ugent.be/gdb/ectocarpus/
Emilhuxl	*Emiliania huxleyi*	NCBI
EuglGrac	*Euglena gracilis*	TBestDB
FontB2	*Fonticula alba*	Broad

Galdsulp	*Galdieria sulphuraria*	NCBI
GlauNost	*Glaucocystis nostochinearum*	TBestDB
Guilthet	*Guillardia theta*	NCBI
GuttMIGQ	*Guttulinopsis vulgaris*	NCBI
JakoLibe	*Jakoba libera*	TBestDB
Klebflac	*Klebsormidium flaccidum*	NCBI
**Lothocea**	***Lotharella oceanica***	
MastBala	*Mastigamoeba balamuthi*	TBestDB
Mesoviri	*Mesostigma viride*	NCBI
Micrpusl	*Micromonas pusilla*	NCBI
Monobrev	*Monosiga brevicollis*	NCBI
Naegleri	*Naegleria gruberi*	NCBI
Nitehyla	*Nitella hylalina*	NCBI
OltmviriA	*Oltmannsiellopsis viridis*	Plant 1KP
OryzSati	*Oryza sativa*	NCBI
Ostrluci	*Ostreococcus lucimarinus*	JGI
Ostrtaur	*Ostreococcus tauri*	NCBI
OxyrMari	*Oxyrrhis marina*	TBestDB
Paratetr	*Paramecium tetraurelia*	NCBI
Paulchro	*Paulinella chromatophora*	Supplemental file of 10.1093/molbev/msq209
Pavl2436	Pavlovales sp. CCMP2436	NCBI SRA
PCBtrin	*Pygsuia biforma*	NCBI
Perkmari	*Perkinsus marinus*	NCBI
Phaetric	*Phaeodactylum tricornutum*	NCBI
Physpate	*Physcomitrella patens*	NCBI
PhysSRA	*Physarum polycephalum*	NCBI SRA
PhytInfe	*Phytophthora infestans*	NCBI
Plasmodi	*Plasmodium falciparum*	NCBI

Porpumbi	*Porphyra umbilicalus*	NCBI SRA
Porpyezo	*Porphyra yezoensis*	NCBI SRA
Prymparv	*Prymnesium parvum*	NCBI
ReclAmer	*Reclinomonas americana*	TBestDB
Rhodsali	*Rhodomonas salina*	NCBI
Roomtrun	*Roombia truncata*	NCBI
SawyMary	*Sawyeria marylandensis*	TBestDB
Selamoel	*Selaginella moellendorffii*	PhytoZomeJGI
Spirprat	*Spirogyra pratensis*	NCBI
Spizpunc	*Spizellomyces punctatus*	Broad
SymbKB8	*Symbiodinium* sp. K8	Medina Lab
Tetrther	*Tetrahymena thermophila*	NCBI
ThalPseu	*Thalassiosira pseudonana*	NCBI
Thectrah	*Thecamonas trahens*	Broad
Trimpyri	*Trimastix pyriformis*	TBestDB
Trypbruc	*Trypanosoma brucei*	NCBI
Ulvaprol	*Ulva prolifera*	NCBI
Volvcart	*Volvox carteri*	NCBI
Welwmira	*Welwitschia mirabilis*	NCBI

PLASTID

ArabPLAS	Arabidopsis thaliana	NCBI
Bigenata	*Bigelowiella natans*	JGI
Bryohypn	Bryopsis hypnoides	NCBI
Chaeglob	Chaetosphaeridium globosum	NCBI
Charvulg	Chara vulgaris	NCBI
ChlaPLAS	Chlamydomonas reinhardtii	NCBI
ChloNC64	*Chlorella* sp. NC64	JGI
Chlovari	Chlorella variabilis	NCBI

Chlovulg	Chlorella vulgaris	NCBI
Cyancald	Cyanidium caldarium	NCBI
CyanCYAN	Cyanophora paradoxa	NCBI
CyanMero	*Cyanidioschyzon merolae*	NCBI
Dunasali	Dunaliella salina	NCBI
EuglGrac	Euglena gracilis	NCBI
Eugllong	Euglena longa	NCBI
Floyterr	Floydiella terrestris	NCBI
Ginkbilo	Ginkgo biloba	NCBI
Gractenu	Gracilaria tenuistipitata-liui	NCBI
**Lothocea**	***Lotharella oceanica***	
Mesoviri	*Mesostigma viride*	NCBI
MicrC299	Micromonas sp. RCC299	NCBI
MicrPLAS	Micromonas pusilla	NCBI
Oedocard	Oedogonium cardiacum	NCBI
Oltmviri	Oltmannsiellopsis viridis	NCBI
OryzPLAS	Oryza sativa	NCBI
Ostrtaur	*Ostreococcus tauri*	NCBI
Physpate	*Physcomitrella patens*	NCBI
Pinuthun	Pinus thunbergii	NCBI
Porpyezo	Porphyra yezoensis	NCBI
Pseuakin	Pseudendoclonium akinetum	NCBI
Scenobli	Scenedesmus obliquus	NCBI
Schileib	Schizomeris leibleinii	NCBI
Selamoel	*Selaginella moellendorffii*	NCBI
