# Supplementary material for: Nucleomorph and plastid genome sequences of the chlorarachniophyte Lotharella oceanica: convergent reductive evolution and frequent recombination in nucleomorph-bearing algae
Source: BMC Genomics. 2014 May 15;15(1):374. doi: 10.1186/1471-2164-15-374 (PMC4035089; doi:10.1186/1471-2164-15-374)

Addtional file 7: RAxML trees inferred from different protein sets

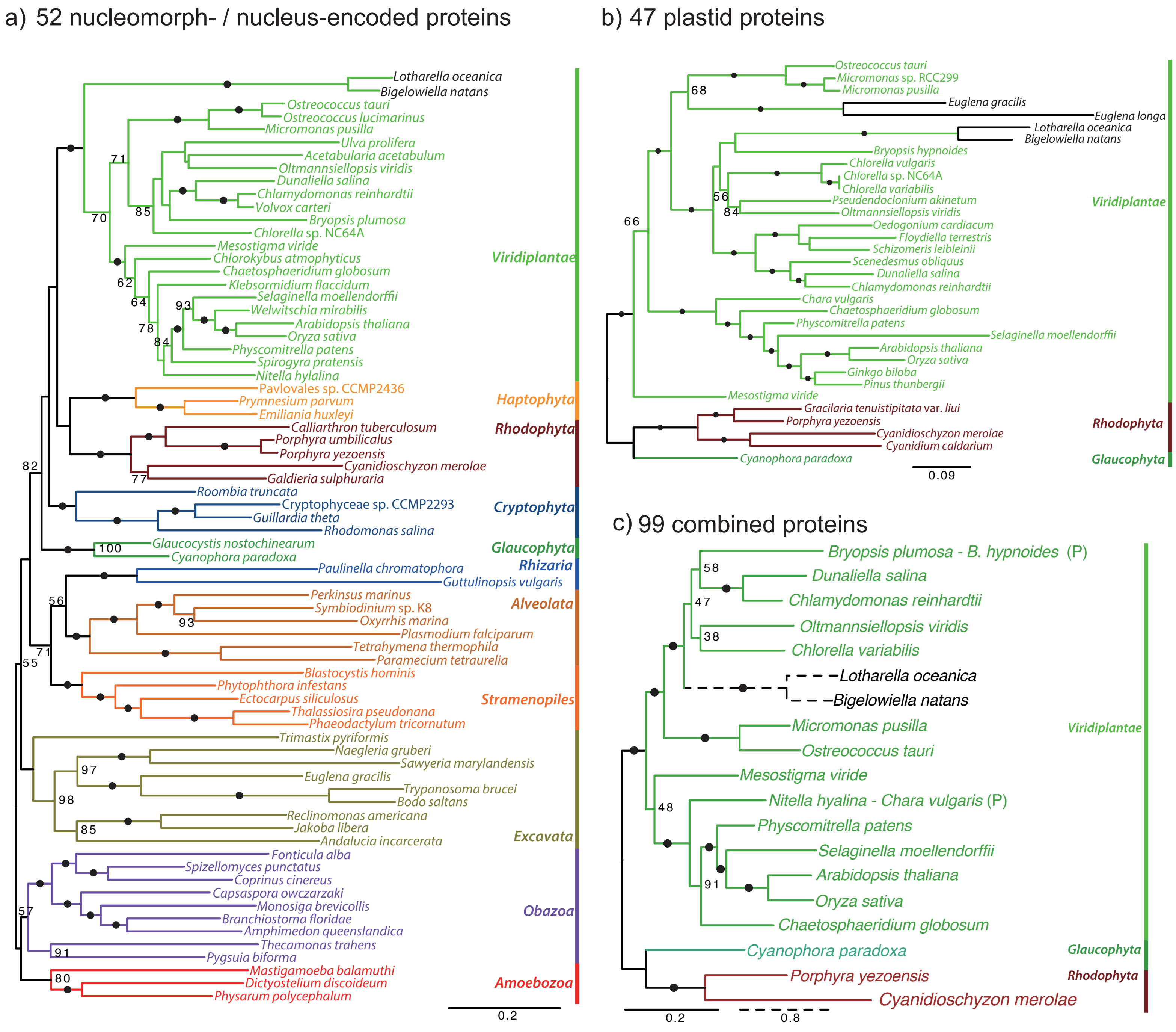

Supplement: Supplementary file 7 — Additional file 7: RAxML trees inferred from different protein sets. (PDF 563 KB) [file 12864_2014_6068_MOESM7_ESM.pdf]
